# Aryl Hydrocarbon Receptor-Dependent Retention of Nuclear HuR Suppresses Cigarette Smoke-Induced Cyclooxygenase-2 Expression Independent of DNA-Binding

**DOI:** 10.1371/journal.pone.0074953

**Published:** 2013-09-27

**Authors:** Michela Zago, Jared A. Sheridan, Parameswaran Nair, Angela Rico de Souza, Imed-Eddine Gallouzi, Simon Rousseau, Sergio Di Marco, Qutayba Hamid, David H. Eidelman, Carolyn J. Baglole

**Affiliations:** 1 Department of Medicine, McGill University, Montreal, Quebec, Canada; 2 Research Institute of the McGill University Health Centre, Montreal, Quebec, Canada; 3 Department of Biochemistry and the Goodman Cancer Centre, McGill University, Montreal, Quebec, Canada; 4 Department of Medicine, McMaster University, Hamilton, Ontario, Canada; National Institute of Health (NIH), United States of America

## Abstract

The aryl hydrocarbon receptor (AhR), a ligand-activated transcription factor that responds to man-made environmental toxicants, has emerged as an endogenous regulator of cyclooxygenase-2 (Cox-2) by a mechanism that is poorly understood. In this study, we first used AhR-deficient (*AhR^−/−^*) primary pulmonary cells, together with pharmacological tools to inhibit new RNA synthesis, to show that the AhR is a prominent factor in the destabilization of *Cox-2* mRNA. The destabilization of *Cox-2* mRNA and subsequent suppression of cigarette smoke-induced COX-2 protein expression by the AhR was independent of its ability to bind the dioxin response element (DRE), thereby differentiating the DRE-driven toxicological AhR pathway from its anti-inflammatory abilities. We further describe that the AhR destabilizes *Cox-2* mRNA by sequestering HuR within the nucleus. The role of HuR in AhR stabilization of *Cox-2* mRNA was confirmed by knockdown of HuR, which resulted in rapid *Cox-2 *mRNA degradation. Finally, in the lungs of *AhR^−/−^* mice exposed to cigarette smoke, there was little *Cox-2* mRNA despite robust COX-2 protein expression, a finding that correlates with almost exclusive cytoplasmic HuR within the lungs of *AhR^−/−^* mice. Therefore, we propose that the AhR plays an important role in suppressing the expression of inflammatory proteins, a function that extends beyond the ability of the AhR to respond to man-made toxicants. These findings open the possibility that a DRE-independent AhR pathway may be exploited therapeutically as an anti-inflammatory target.

## Introduction

Cigarette smoke is the leading cause of preventable death worldwide and is the primary risk factor for the top three mortalities: cardiovascular disease (CVD), cancer and respiratory disease, which includes chronic obstructive pulmonary disease (COPD). COPD affects some 200 million people worldwide [1] and is estimated to become the third leading cause of death within the next decade [2]. COPD is characterized by progressive airflow limitation that is not fully reversible and is associated with chronic inflammation. Cigarette smoke incites and perpetuates this inflammatory response by inducing pro-inflammatory mediator production (lipids, chemokines and cytokines). We recently identified that the aryl hydrocarbon receptor (AhR), a receptor/transcription factor that is highly expressed in the human lung [3], is a novel and potent suppressor of cigarette smoke-induced inflammation [4], [5]. The AhR is a member of the basic helix-loop-helix Per-Arnt-Sim (bHLH-PAS) transcription factor family that is well-known to respond to man-made xenobiotics such as 2,3,7,8-tetrachlorodibenzo-*p*-dioxin (TCDD; dioxin) and related planar aromatic hydrocarbons [6]. In the absence of ligand, the AhR is found in the cytoplasm complexed with chaperone proteins, including a dimer of heat shock protein 90 (HSP90) and the immunophilin hepatitis B virus X-associated protein 2 (XAP2) [7], [8], [9]. After ligand binding, the AhR translocates to the nucleus, dissociates from these chaperones and forms a heterodimer with the AhR nuclear transporter (ARNT). This AhR:ARNT complex then binds to a dioxin responsive element (DRE) and initiates the transcription of genes that comprise the AhR gene battery, including cytochrome P450 (CYP) enzymes.

Numerous early-response genes encoding inflammatory mediators such as cyclooxygenase-2 (*Cox-2*) also contain a DRE in the promoter region and can be increased due to AhR activation by dioxin [10], [11], [12]. COX-2 is an inducible enzyme that catalyzes the transformation of arachidonic acid into thromboxanes and prostaglandins (PG) such as PGE_2_. COX-2 is robustly increased by cigarette smoke exposure [13] and is elevated in patients with inflammation-associated diseases including COPD [14], [15]. Although cigarette smoke contains components capable of activating the AhR, including benzo[*a*]pyrene (B[*a*]P) [16], our published data demonstrate that expression of the AhR suppresses COX-2 protein expression and PG production due to cigarette smoke exposure [4]. Interestingly, AhR expression was associated with a rapid, significant but transient increase in *Cox-2* mRNA upon smoke exposure. Despite this increase in *Cox-2* mRNA, there is little COX-2 protein expression [4], suggesting that the AhR suppress COX-2 protein by post-transcriptional regulatory mechanisms.

Post-transcriptional regulation of protein expression is an adaptive mechanism that is crucial in regulating the timing and the amount of inflammatory proteins. Although the *Cox-2* gene is transcriptionally-controlled, the level of COX-2 protein is determined in large part by changes in the half-life of the mRNA. Thus, there is often a poor correlation between *Cox-2* mRNA and protein levels because *Cox-2* mRNA is rapidly degraded. The instability of *Cox-2* mRNA is due to the presence of adenylate- and uridylate- rich element (ARE) in the 3′-untranslated region (UTR) [17], which can be bound by proteins that can alter *Cox-2* mRNA stability and translation [18]. RNA-binding proteins that interact with the *Cox-2* ARE include the CELF/Bruno-like family member CUGBP2 [19] and the embryonic lethal abnormal vision (ELAV)-like protein Human antigen R (HuR) [20]. HuR is a ubiquitous RNA-binding protein that is abundantly localized to the nucleus, where it is first interacts with *Cox-2* mRNA. HuR subsequently shuttles between the nucleus and cytoplasm upon stimulation. It is believed that cytoplasmic localization is important for the mRNA-stabilizing effects of HuR [21], [22], [23]. Whether the AhR regulates *Cox-2* mRNA stability by controlling HuR expression or localization is not known.

Herein, we used lung cells devoid of AhR expression, together with our established *in vitro* and *in vivo* models of cigarette smoke exposure [4], [5], [24] and show that the AhR-dependent retention of nuclear HuR is responsible for the destabilization of *Cox-2* mRNA by a mechanism that was independent of AhR:DNA binding activity. Therefore, despite its dubious distinction as a transcriptional regulator of toxicological outcomes, we propose that the AhR plays an important role in the suppression of inflammation that extends beyond its ability to respond to man-made toxicants.

## Materials and Methods

### Chemicals

All chemicals were purchased from Sigma (St. Louis, MO) unless otherwise indicated. Actinomycin D (ActD) was purchased from Biomol (Plymouth Meeting, PA). Recombinant mouse IL-1β was purchased from R&D Systems (Minneapolis, MN). CH-223191 (1-Methyl-N-[2-methyl-4-[2-(2-methylphenyl) diazenyl] phenyl-1H-pyrazole-5-carboxamide) was from Tocris Bioscience (Minneapolis, MN).

### Cell Culture

#### Mouse lung fibroblasts

Primary lung fibroblasts were generated from *AhR^+/+^*, *AhR* heterozygous (*AhR^+/−^*) and *AhR^−/−^* C57BL/6 mice (Jackson Laboratory, Bar Harbor, ME) [25] and cultured under standard conditions [4], [24]. Lung fibroblasts were also generated from a novel lineage of mice harboring a mutant AhR that is incapable of binding to DNA (referred to hereafter as *AhR^DBD/DBD^*) [26], a kind gift of Dr. Chris Bradfield (University of Wisconsin); lung fibroblasts from littermate heterozygotes (*AhR^DBD/B6^*) are used as corresponding controls. Unless otherwise indicated, all pulmonary fibroblasts were plated at a density of 10,000 cells/cm^2^ and most experiments were conducted using fibroblasts generated from at two different *AhR^−/−^* mice. Lung fibroblasts from wild-type or heterozygous mice do not exhibit any difference in the ability to be activated by AhR ligands and are used interchangeably as AhR-expressing cells [4], [24].

#### Human lung fibroblasts

Primary lung fibroblasts were cultured and characterized as previously described [25] from lung tissue derived from individuals undergoing lung resection surgery for suspected lung cancer at McMaster University. Only tissue from disease-free regions was used for the derivation of fibroblasts and all subjects were reported never-smokers. This study was approved by the Research Ethics Board of St Joseph’s Healthcare Hamilton and all patients gave written informed consent. All fibroblast strains were used at the earliest possible passage.

#### Hepa.2DLuc.3A4 (Hepa.2Dluc)

Mouse hepatoma cells stably transfected with the luciferase reporter plasmid p2DLuc, which contains two copies of the DRE_D_ consensus sequence [27], [28] and is thus a direct measure of classic AhR activation. Derivation of Hepa.2Dluc cells were previously described [27] and were a kind gift of Dr. Tom Gasiewicz (University of Rochester). Hepa.2Dluc cells were cultured in minimum essential media (MEM) supplemented with 2 mM glutamine (Invitrogen, Carlsbad, CA), 10% fetal bovine serum (FBS) (Hyclone Labs, Logan, UT) and antibiotics/antimycotics (penicillin G, streptomycin and amphotericin; Invitrogen, Carlsbad, CA).

#### Lung epithelial cells

MLE-12 cells, a distal bronchiolar and alveolar epithelial cell line (ATCC, Manassas, VA) [29], were cultured in HITES medium (50∶50 DMEM: Ham’s F12) supplemented with 2% FBS, 2 mM L-Glutamine, 10 mM HEPES, 1∶100 Insulin-Transferrin-Selenium supplement (Invitrogen) and antibiotics/antimycotics.

### In Vivo Cigarette Smoke Exposure

Age- and gender-matched *AhR^−/−^* or *AhR^+/−^* littermate controls were exposed to cigarette smoke as previously described [5]. Briefly, research cigarettes (University of Kentucky, Lexington, KY) were smoked according to the Federal Trade Commission protocol (1 puff/minute/cigarette of 2 seconds duration and 35-ml volume). Control and *AhR^−/−^* mice were exposed to cigarette smoke for 5 days a week for 2 and 4 weeks (sub-chronic exposures). Daily exposures were for one hour, twice daily at four-hour intervals. Control mice were exposed to filtered air. As we have previously published that there is no difference between wild-type (*AhR^+/+^*) C57BL/6 and *AhR^+/−^* mice [5], *AhR^+/−^* mice are used for the *in vivo* studies. All animal procedures were approved by the McGill University Animal Care Committee (Protocol Number: 5933) and were carried out in accordance with the Canadian Council on Animal Care. Following exposure, mice were anesthetized with Avertin (2,2,2-tribromoethanol, 250 mg/kg i.p.; Sigma-Aldrich) and euthanized by exsanguination. The lungs were immediately excised, the left lung inflated with OCT and snap**-**frozen in liquid nitrogen. A portion of the right lung was immediately placed in RNAlater® (Qiagen, Toronto ON) or frozen in liquid nitrogen for further protein analysis.

### Preparation of Cigarette Smoke Extract (CSE)

Research grade cigarettes (2R3F) with a filter were obtained from the Kentucky Tobacco Research Council (Lexington, KT) and CSE generated as previously described [4], [30], [31], [32]. Briefly, CSE was prepared by bubbling smoke from two cigarettes into 20 ml of serum-free MEM, the pH adjusted to 7.4, sterile- filtered with a 0.45-µm filter (25-mm Acrodisc; Pall Corp., Ann Arbor, MI) and was used within 30 minutes of preparation. An optical density of 0.65 (320 nm) was considered to represent 100% CSE [4], [24] which was diluted to the appropriate concentration in serum-free MEM.

### Western Blot

Fibroblasts were grown to sub-confluence and cultured in serum-free MEM for 24 hours before being treated with CSE for the indicated times. Total cellular protein was prepared using 1% IGEPAL lysis buffer [31]; nuclear and cytoplasmic fractions were prepared using a nuclear extract kit (Active Motif, Carlsbad, CA). Five to ten µg of cellular proteins were fractionated on SDS-PAGE gels, electroblotted onto PVDF membranes and antibodies against AhR (1∶5000; Enzo Life Sciences, NY, USA), COX-2 (1∶1000, Cayman Chemical, Michigan, USA), HuR (1∶2000), CUGBP2, CYP1A1, CYP1B1 (1∶500, Santa Cruz, Santa Cruz, CA) and actin (1∶20,000; Millipore, MA, USA) were used to assess changes in protein levels by enhanced chemiluminescence (ECL). Protein bands were visualized using a gel documentation system (Alpha Innotech, San Leandro, CA).

### Analysis of Gene Expression

Total RNA was harvested and quantification was conducted on a Nanodrop 1000 spectrophotometer (Thermo Fisher Scientific, Wilmington, DE). Reverse transcription of total RNA was carried out using iScript II™ Reverse Transcription Supermix (Bio-Rad Laboratories, Mississauga, ON). Quantitative PCR was then performed by addition of 1 µl cDNA and 0.5 µM primers with SsoFast™ EvaGreen® (Bio-Rad). The primer sequences were: *Cox-2*- TGCCTGGTCTGATGATGTATGCCA (f) and AGTAGTCGCACACTCTGTTGTGCT (r); *Cyp1a1*- CCTTACCAAGTGCTAGGATACAGTCATAGA (f) and CAGTAAAGAAGAGAGACCAAGAGCTGAT (r); *Cyp1b1*-AAAATGTAAAGACCAGAAGTC CTCCTACC (f) and AGAAAGCCTCATCCAGGGCTATAAA (r) and *β-actin*- CTACAATGAGCTGCGTGTG (f) and TGGGGTGTTGAAGGTCTC (r). PCR amplification was performed using a CFX96 Real-Time PCR Detection System (Bio-Rad). Melt curve analysis was performed to ensure that nonspecific products were absent. The fluorescence detection threshold was set above the non-template control background within the linear phases of PCR amplifications and the cycle threshold (Ct) of each reaction was detected. Gene expression was analyzed using the ΔΔCt method and results are presented as fold-change normalized to housekeeping gene (*β-actin*).

### Determination of *Cox-2* mRNA Stability

*AhR^−/−^* and *AhR*-expressing lung fibroblasts were cultured in 6-well culture plates until near confluence and switched to serum-free media for 24 hours. Then the fibroblasts were exposed to 1% CSE for 3 hours followed by treatment with ActD (1 µg/ml), an inhibitor of RNA synthesis [33], for 30 minutes or for 1 or 3 hours. Total RNA was harvested and qPCR performed as described above to determine the remaining levels of mRNA. In separate experiments, *AhR^DBD/DBD^* and *AhR^DBD/B6^* cells were also exposed to ActD with and without CSE. To determine if inhibition of AhR activity altered *Cox-2* mRNA stability, *AhR^DBD/DBD^* and *AhR^DBD/B6^* cells were treated with CH-223191 together with ActD and 1% CSE and *Cox-2 *mRNA levels assessed. The concentration of ActD used in these experiments did not affect cell viability (data not shown). To verify inhibition of *Cox-2* mRNA synthesis, in separate experiments, *AhR^+/−^* fibroblasts were pretreated with 1 µg/ml of ActD followed by treatment with IL-1β (10 ng/ml) for 6 hours.

### Reporter Gene Assay

Hepa.2Dluc cells were seeded in six-well plates (4×10^5^ cells/well) and allowed to grow overnight. Cells were then pretreated with vehicle (DMSO), 1 or 10 µM CH-223191 for 1 hour following by 6 hour treatment with 1 µM B[a]P. After treatments, cell lysates were collected and luciferase activity measured using the Luciferase Assay System (Promega, Madison, WI) and read on the Infinite M1000 microplate reader (Tecan, Mannedorf, Switzerland).

### Immunofluorescence

#### Primary Cell Culture

Fibroblasts were seeded on 8-well glass chamber slides at a density of 1×10^4^ cells/well and allowed to adhere for 24 h. Following serum starvation for 24 h, the cells were treated with media only, 1% CSE or B[a]P for 1, 4 or 24 hours to assess HuR and CUGBP2 localization or COX-2 expression. Following treatments, the cells were washed once with PBS/Tween, permeabilized/fixed using 3% H_2_O_2_/methanol for 10 min, and blocked with Universal Blocking Solution (Dako, ON, CA) for 30 minutes at room temperature. The antibodies against HuR, CUGBP2 (Santa Cruz) and COX-2 (Cayman) were diluted 1∶200 in PBS/bovine serum albumin (BSA) and incubated overnight at 4°C. Levels of non-specific staining were assessed by incubating cells under identical conditions using the isotype- matched non-immune antibody (Santa Cruz) at the same concentration or by omission of the primary antibody. In all cases, the level of non-specific staining was negligible (data not shown). Alexa Fluor-488 anti-mouse or anti-rabbit IgG antibody was used for secondary binding (1∶1000) and incubated for 1 hour at room temperature. Slides were then mounted in ProLong® Gold Anti Fade (Invitrogen), viewed on an Olympus BX51 fluorescent microscope (Olympus, Ontario, Canada) and photographed using a Retiga 2000R Camera with ImagePro Plus software. Fluorescent images of nuclei are visualized by Hoechst staining (1∶2000, Molecular Probes). All photographs were taken at the same time with identical image settings. For quantification, positive and negative cells were counted in each picture taken and recorded per randomly-selected field (minimum of five separate fields per experiment). Cells were considered positive based on fluorescence intensity within the cytoplasm. Positive cells were compared to the total counted cells for each individual experiment and expressed as a percentage of the total cells present.

#### Mouse lung tissue

OCT-embedded lung from air- or CS-exposed mice were sectioned, fixed in 70% ethanol for 3 minutes and permeabilized in 0.5% PBS/Tween20 for 10 at room temperature (RT). Then, the sections were blocked with the Universal Blocking Solution (Dako, ON, CA) for 30 minutes. For detection of HuR and COX-2/vimentin, the lung sections were incubated with goat anti-mouse HuR (1∶300) or COX-2/vimentin antibodies (1∶200/1∶100) (Cayman) for 1 hour at RT. After rinsing with PBS/Tween, the sections were then incubated with Alexa 555-conjugated rabbit anti-mouse IgG (vimentin and HuR) and Alexa-488-conjugated donkey anti-rabbit IgG (COX-2) (Molecular Probes Inc., ON, CA), diluted at 1∶1000 in Dako antibody diluent for 1 hour. The sections were then cover-slipped with ProLong® Gold Anti Fade mounting medium (Invitrogen). Fluorescent images for COX-2/vimentin were detected via a fluorescence microscope (Olympus BX51TF) whereas HuR localization was assessed by a Laser Scanning Microscope-LSM 78 (RI-MUHC Imaging Facility, McGill University, Montreal, Canada).

### Enzyme Immunoassay

Equivalent numbers of *AhR^DBD/DBD^* and *AhR^DBD/B6^* fibroblasts were allowed to reach confluence and serum-starved for 24 hours prior to stimulation with CSE for varying time-points. Controls included incubation with serum-free MEM. The resulting amount of PGE_2_ in the cell culture supernatant was determined via specific enzyme immunoassay (EIA) as described previously [4], [13].

### siRNA Knockdown Studies

*AhR^+/+^* and *AhR^−/−^* lung fibroblasts were grown to approximately 60–80% confluence, after which the cells were transiently transfected with 60 nM siRNA against HuR or with control siRNA. Two different siRNA targeting HuR were utilized for these experiments (siRNA-1: Santa Cruz, CA; siRNA-2: Dharmacon, ON). Separate controls for each HuR siRNA were also used. Transfections were performed according to the manufacturer’s instructions for 24–48 hours. During the transfection process, cells were pre-treated with 1% CSE for 3 hours followed by 1 µg/ml ActD for 30 minutes or for 1 or 3 hours. Then, total RNA was isolated as described above and qPCR performed for *Cox-2.* Knockdown of HuR was confirmed by western blot analysis.

### Statistical Analysis

Statistical analysis was performed using JMP®8 (SAS Institute, Cary, NC). An analysis of variance (ANOVA) with Tukey-Kramer post-hoc test was used to assess differences between treatment groups of more than two. Results are expressed as the mean ± SEM. In all cases, a p value<0.05 is considered statistically significant.

## Results

### Antagonism of the AhR by CH-223191 Promotes CSE-induced COX-2 Protein Expression in Primary Lung Fibroblasts

**Figure 1:**
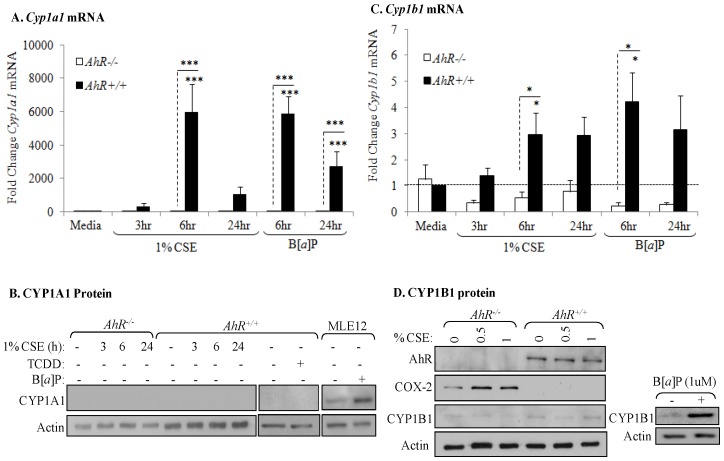
AhR activation by CSE does not increase COX-2 protein. *AhR^−/−^* and *AhR^+/+^* lung fibroblasts were exposed to CSE or B[*a*]P (1 µM) for 3, 6 or 24 hours and whole cell lysates collected for protein or RNA analysis. (A) There was a significant increase in *Cyp1a1* mRNA in response to both CSE and B[*a*]P for 6 hours only in *AhR^+/+^* cells (***p<0.0001). Results are expressed as the mean ± SEM of 3–6 independent experiments. (B) Basal levels of CYP1A1 protein were not detectable in primary lung fibroblasts. CYP1A1 was not increased by CSE or the AhR ligand TCDD. MLE-12 cells express basal CYP1A1 that was further increased by B[*a*]P treatment. Western blot is representative of three experiments. (C) There was significantly more *Cyp1b1* mRNA in lung fibroblasts exposed to 1% CSE or B[*a*]P compared to *AhR^−/−^* cells. Results are expressed as the mean ± SEM of 3–8 independent experiments. (D) There is no CYP1B1 protein induction by CSE exposure for 24 hours; note the increase in COX-2 protein only in *AhR^−/−^* fibroblasts. B[*a*]P increased CYP1B1 protein expression in *AhR^+/+^* fibroblasts. Representative western blot is shown.

Our published data show that CSE robustly increases COX-2 protein expression in AhR-deficient cells with no induction of *Cox-2* mRNA [4], supporting a post-transcriptional regulatory role for the AhR in regulating COX-2 expression. Now, we first sought to determine if AhR activation by CSE was necessary to suppresses COX-2 expression. Utilizing cytochrome P450 (CYP) induction as a well-defined marker of AhR activation [34], we show there was a significant increase in *Cyp1a1* mRNA in *AhR^+/+^* cells by CSE, similar to that induced by the classic AhR ligand B[*a*]P (Figure 1A). Consistent with previous reports [35], [36], there was no CYP1A1 protein in lung fibroblasts (Figure 1B). *Cyp1b1* expression, the predominant CYP isoform expressed by fibroblasts [24], [35], [36], was increased by CSE and B[*a*]P in *AhR^+/+^* cells (Figure 1C). CYP1B1 protein increased only in B[*a*]P-exposed control cells but not those exposed to CSE (Figure 1D). Note the increase in COX-2 protein expression is only in the *AhR^−/−^* fibroblasts exposed to CSE, consistent with our published data [4].

**Figure 2:**
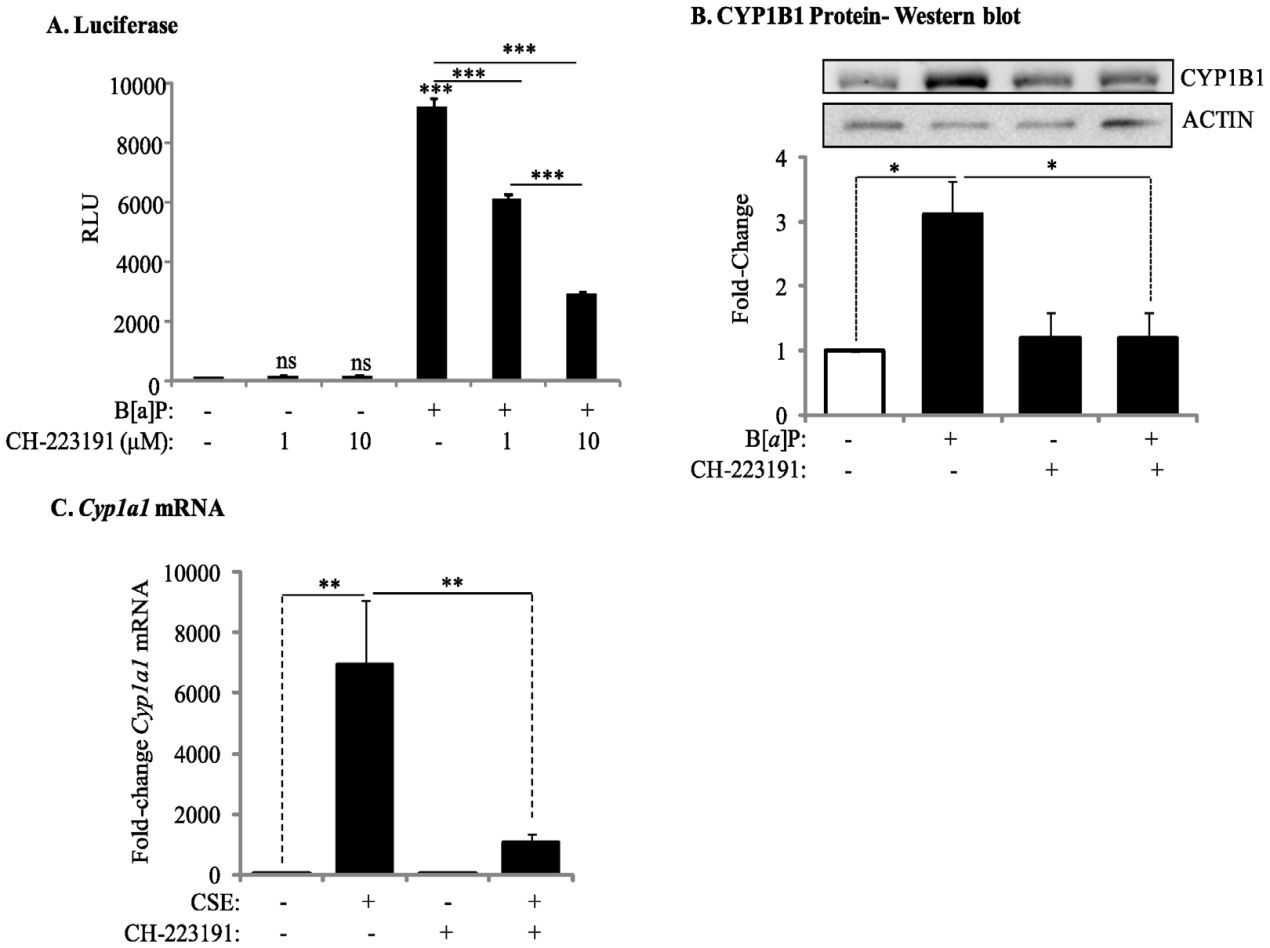
Inhibition of AhR activity by the pharmacological antagonist CH-223191. (A) Hepa.2Dluc cells were pre-treated with CH-223191 (10 µM) for one hour followed by treatment with B[a]P for 6 hours and cell lysates collected for luciferase activity. There was no induction in RLU when Hepa.2Dluc cells were treated with CH-223191 alone (ns = not significant compared to DMSO). There was a significant increase in RLU when Hepa.2Dluc were exposed to B[*a*]P (***p<0.0001 compared to DMSO). Pretreatment with CH-223191 dose-dependently inhibited luciferase activity elicited by B[*a*]P alone. Results are representative of two independent experiments and data are expressed as mean ± SEM. (B) There was a significant increase in CYP1B1 protein in mouse lung fibroblasts exposed to B[*a*]P; this increase was reduced by CH-223191. Results are expressed as the mean ± SEM of 3 independent experiments (*p<0.05); representative western blot is shown. (C) CSE-induced *Cyp1a1* mRNA is significantly attenuated by CH-223191 in AhR-expressing mouse lung cells. Results are expressed as the mean ± SEM of 4 independent experiments (**p<0.01).

To further evaluate if the ability of the AhR to suppress COX-2 protein induction by CSE requires AhR activation, we used the pharmacological AhR antagonist CH-223191, which binds to the AhR and prevents ligand-induced AhR translocation to the nucleus and subsequent DRE-mediated transcription. Using Hepa.2DLuc cells, a mouse hepatoma cell line stably transfected with the AhR reporter plasmid p2Dluc [27], [28], we determined that there was no significant change in DRE-mediated transcription (Figure 2A), indicating that CH-223191 does not exhibit any agonist activity [37]. Furthermore, CH-223191 dose-dependently antagonized B[*a*]P-induced AhR activation (Figure 2A). Exposure of *AhR^+/−^* cells to B[*a*]P increased CYP1B1 protein expression, which was significantly reduced by CH-223191 (Figure 2B). As CH-223191 may exhibit ligand-selective antagonism of the AhR [38], we confirmed that the significant increase in *Cyp1A1* mRNA in response to CSE was also significantly attenuated by CH-223191 (Figure 2C).

**Figure 3:**
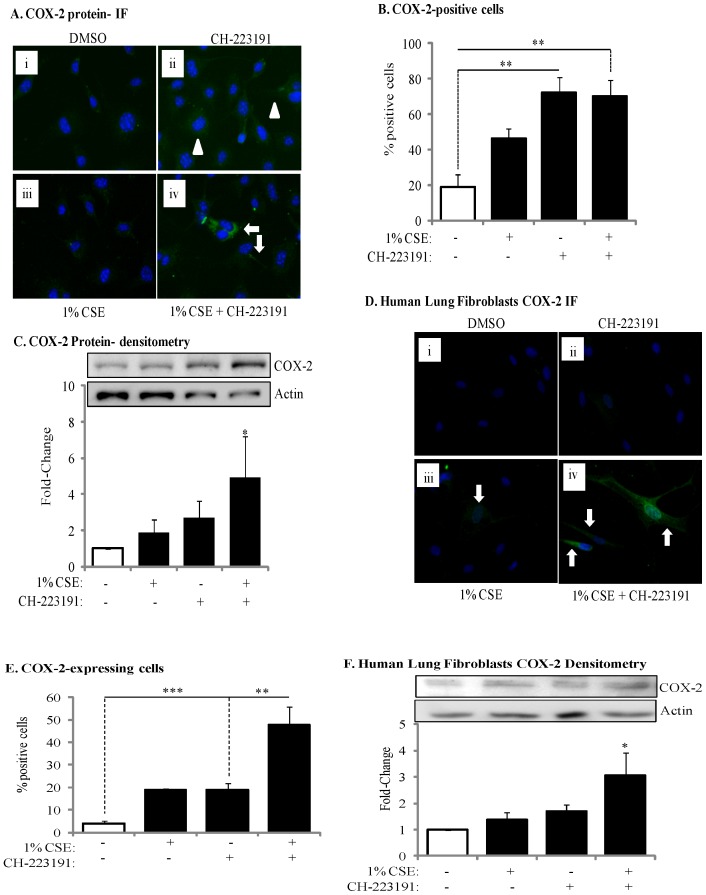
Inhibition of AhR activity augments CSE-induced COX-2 protein expression. (A) There was a slight but detectible increase in COX-2 in the AhR^+/−^ fibroblasts treated with CH-223191 alone (panel *ii*, arrowheads). When cells were exposed to both CSE and CH-223191, there was a strong induction of COX-2 (panel *iv*, arrows). Magnification = 40×. (B) There was a significant increase in the percentage of COX-2-positive cells in response to CH-223191 with or without CSE. Results are expressed as the mean ± SEM for 5 randomly-selected fields per triplicate experiment (**p<0.01). (C) There was a significant induction (fold-change: 4.93±2.4) in COX-2 protein expression when AhR-expressing cells were exposed to CH-223191 and 1% CSE compared to exposure to DMSO alone. Results are expressed as the mean ± SEM of 3 independent experiments. Representative western blot is shown. (D) Human lung fibroblasts- There was a slight but detectable increase in CSE-exposed human lung fibroblasts (panel *iii*). When cells were exposed to both 1% CSE and CH-223191, there was a stronger induction of COX-2 (panel *iv*, arrows). Magnification = 40×. (E) There was a significant increase in the percentage of COX-2-positive human lung fibroblasts in response to CH-223191 and exposed to CSE. Results are expressed as the mean ± SEM for 5 randomly-selected fields per triplicate experiment (**p<0.01; ***p<0.001). (F) There was a significant induction in COX-2 protein expression in human lung fibroblasts exposed to 1% CSE in conjunction with CH-223191 (3.1±0.84; *p<0.05 compared to both media control and 1% CSE alone). Representative western blot is shown. Results are expressed as the mean ± SEM of experiments utilizing fibroblasts from three different individuals.

We then evaluated if inhibition of AhR activity by CH-223191 would affect COX-2 protein expression. Following exposure of AhR-expressing lung fibroblasts to 1% CSE for 24 hours, there was no increase in the expression of COX-2 protein (Figure 3A, 3B and 3C). When AhR activity was inhibited with CH-223191, concurrent with exposure to 1% CSE, there was a significant (4.9-fold) increase in COX-2 protein levels (Figure 3B and 3C). We also utilized primary lung fibroblasts derived from healthy, non-smoking adults. When human lung fibroblasts were exposed to 1% CSE, together with CH-223191, there was a marked and significant increase in COX-2 (Figure 3D and 3E). Densitometric analysis of COX-2 western blots also revealed a significant increase in COX-2 protein expression when AhR activity is inhibited with CH-223191 and the cells are exposed to 1% CSE (Figure 3F). Thus, inhibition of AhR activity potentiates CSE-induced COX-2 protein expression.

### The Ability of the AhR to Attenuate CSE Induction of COX-2 Protein Expression and PG Production does not Require a Functional DNA Binding Domain

**Figure 4:**
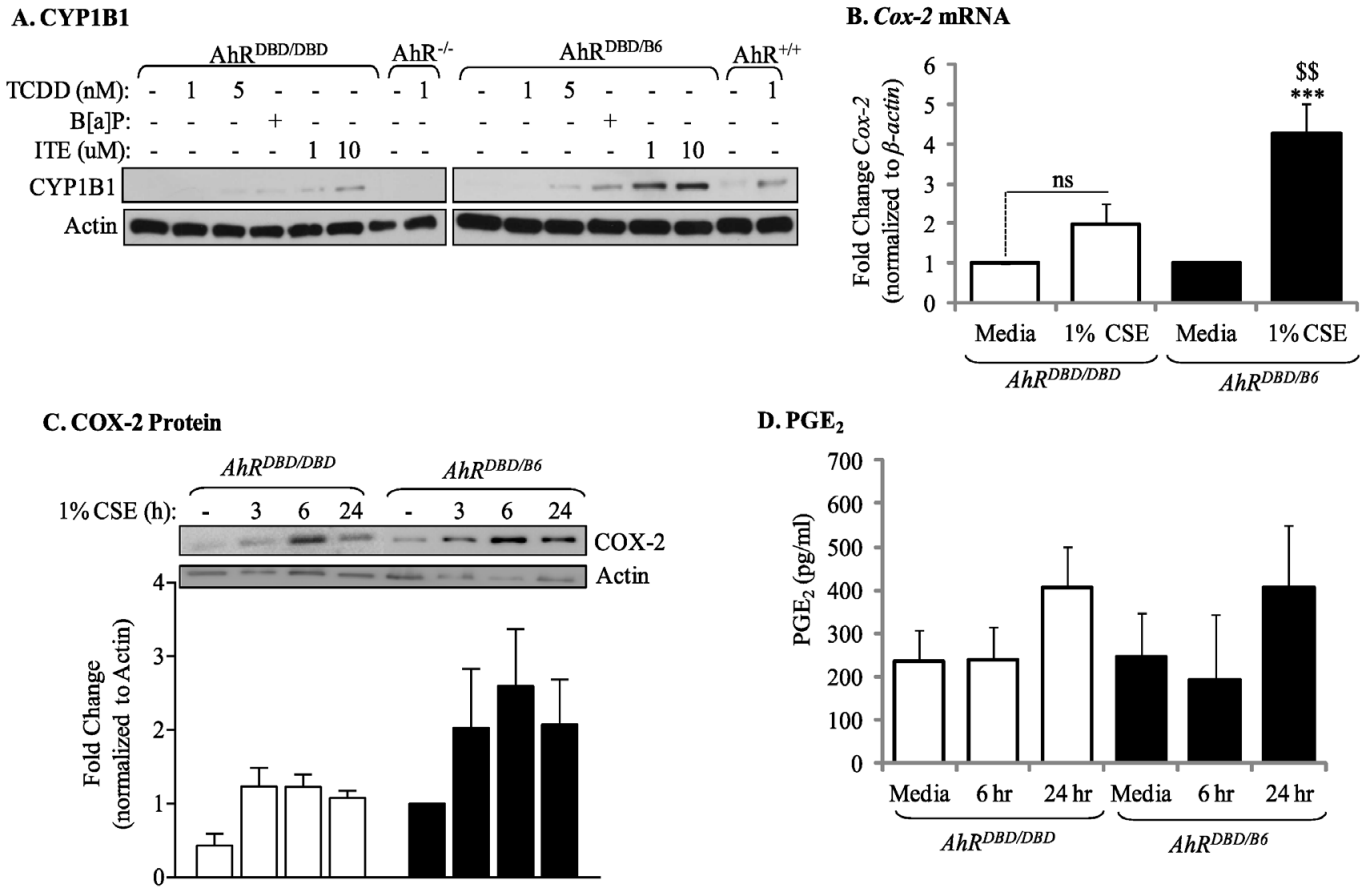
Suppression of CSE-induced COX-2 protein expression and PGE_2_ production does not require DNA binding activity of the AhR. Lung fibroblasts were generated from *AhR^DBD/DBD^* or *AhR^DBD/B6^* mice and treated with AhR ligands (TCDD, ITE and B[*a*]P) or 1% CSE and cellular RNA or protein was collected for qPCR/western blot analysis. Cell culture supernatant was assayed for PGE_2_ production. (A) *AhR^DBD/DBD^* and *AhR^DBD/B6^* lung fibroblasts exposed to AhR ligands (TCDD, B[*a*]P or ITE) induce less CYP1B1 protein expression compared to lung fibroblasts from mice expressing one copy of the wild-type AhR. TCDD-exposed AhR^+/+^ and AhR^−/−^ cells are included for comparison; note the lack of induction in the AhR^−/−^ fibroblasts. (B) *Cox-2* mRNA- There was a slight but not statistically significant increase in *Cox-2* mRNA in *AhR^DBD/DBD^* cells (fold-change was 1.97±0.54; p = 0.5 compared to media-only). There was a significant increase in *Cox-2* mRNA in *AhR^DBD/B6^* fibroblasts in response to CSE (4.3±0.8; ***p<0.001 compared to media-only; ^$$^p<0.01 compared to CSE-exposed *AhR^DBD/DBD^* cells). Results are expressed as mean ± SEM of 5 independent experiments. (C) There was a slight, but not significant, induction of COX-2 protein in response to 1% CSE. The relative level of induction in COX-2 protein was similar between *AhR^DBD/DBD^* and *AhR^DBD/B6^* cells. Results are expressed as mean ± SEM of 5 independent experiments. Representative western blot is shown. (D) Baseline PGE_2_ levels did not differ between *AhR^DBD/DBD^* (235±73 pg/ml) and *AhR^DBD/B6^* (247±99 pg/ml) cells. Exposure to 1% CSE did not significantly increase the concentration of PGE_2_ in either *AhR^DBD/DBD^* or *AhR^DBD/B6^* lung fibroblasts. Samples were run in duplicate and the results are expressed as mean ± SEM of 3–6 independent experiments.

The ability of the AhR to suppress inflammation may be DRE-independent [39]. To determine whether the suppression of cigarette smoke-induced COX-2 protein by the AhR requires DRE binding, we utilized primary lung fibroblasts derived from mice which express an AhR that can bind ligand and translocate to the nucleus, but is incapable of binding the DRE due to a mutation in the AhR DNA-binding domain [26]. We first confirmed that these AhR mutant cells (referred to *AhR^DBD/DBD^*) poorly increase CYP1B1 expression in response to classic AhR ligands (Figure 4A) before evaluating COX-2 expression with CSE. In *AhR^DBD/B6^* cells, there was a significant increase in *Cox-2* mRNA when cells were exposed to CSE for 3 hours (Figure 4B, *black bars*). However, CSE failed to significantly increase *Cox-2 *mRNA in *AhR^DBD/DBD^* fibroblasts (Figure 4B, *open bars*), similar to our published data in *AhR^−/−^*cells [4]. However, the level of COX-2 protein expression in CSE-exposed *AhR^DBD/DBD^* fibroblasts was similar to *AhR^DBD/B6^* cells (Figure 4C). We then determined that downstream PGE_2_ production was not significantly different between *AhR^DBD/DBD^* or *AhR^DBD/B6^* pulmonary fibroblasts (Figure 4D). Collectively, these data support that the ability of the AhR to suppress CSE-induced COX-2 protein expression requires AhR activity but is independent of its DNA-binding abilities.

### Transient Expression of CSE-induced *Cox-2* mRNA is due to AhR-dependent mRNA Destabilization

**Figure 5:**
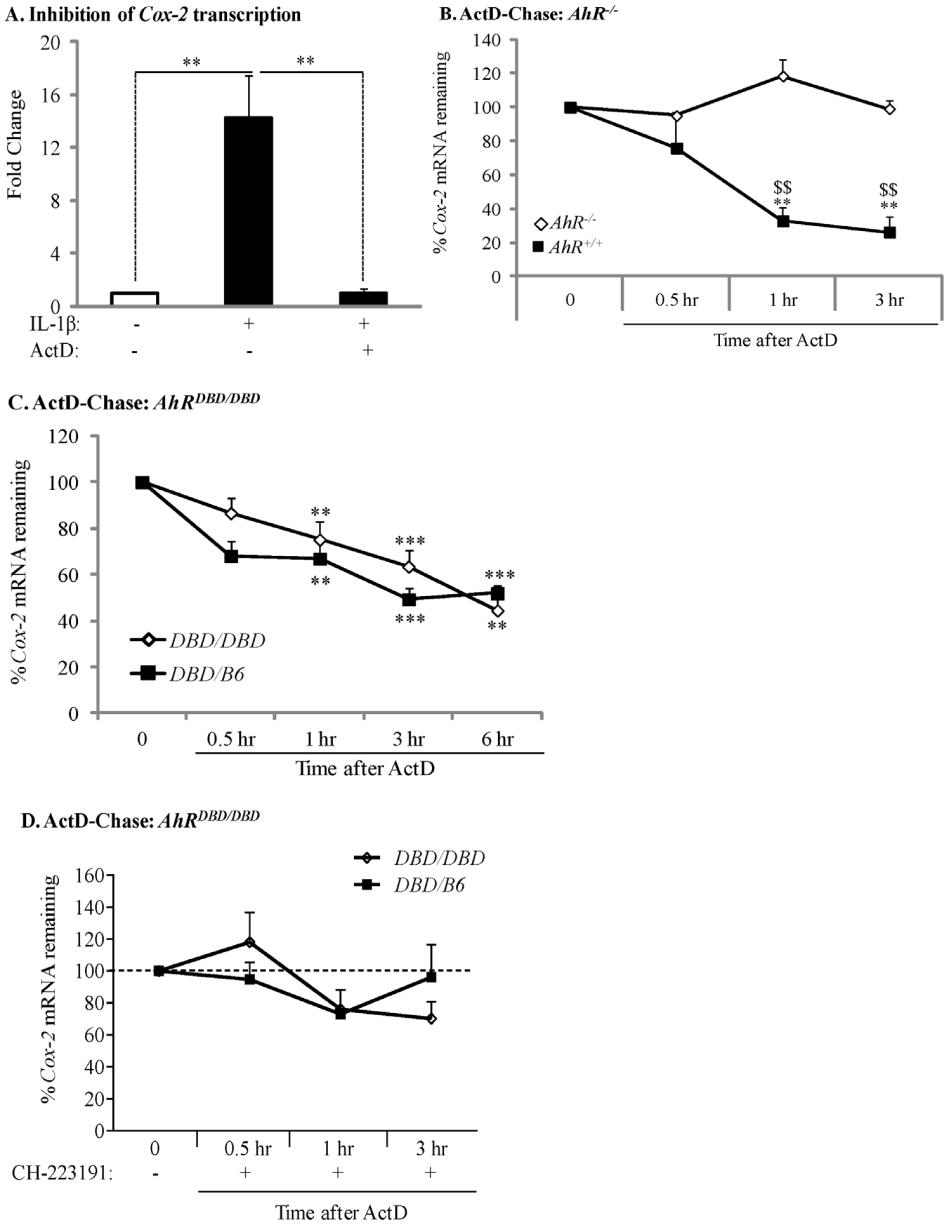
Transient expression of CSE-induced *Cox-2* mRNA is due to AhR-dependent mRNA destabilization. (A) In *AhR^+/−^* lung fibroblasts, there was a significant induction of *Cox-2* mRNA by IL-1β (**p<0.05). Induction of *Cox-2* mRNA was completely blocked by ActD (**p<0.05 compared to IL-1β-treated). Results are expressed as mean ± SEM of normalized *Cox-2* levels and represent results from 3 independent experiments. (B) *AhR^−/−^* and *AhR^+/+^* lung fibroblasts were exposed to 1% CSE for 3 hours and then exposed to ActD (1 µg/ml) for the indicated time-points. *Cox-2* levels were set to equal 100% after CSE exposure for three hours and are expressed as percentage (%) of *Cox-2* mRNA remaining. *Cox-2* mRNA expression remained relatively unchanged in *AhR^−/−^* lung cells whereas there was a rapid and significant decline in *Cox-2* mRNA in *AhR^+/+^* cells after exposure to ActD (**p<0.01 compared to CSE-only exposed control cells; ^$$^p<0.01 compared to *AhR^−/−^* lung at the respective time-point after ActD treatment). Results are expressed as mean ± SEM of normalized *Cox-2* levels and represent data from 4 independent experiments. (C) *AhR^DBD/DBD^* and *AhR^DBD/B6^* lung fibroblasts to 1% CSE for 3 hours followed by exposure to ActD (1 µg/ml) for the indicated time-points. There was a rapid and significant decline in *Cox-2* mRNA in both *AhR^DBD/DBD^* and *AhR^DBD/B6^* lung cells after exposure to ActD for 1, 3 or 6 hours (**p<0.01 and ***p<0.001 compared to CSE-only exposed control cells (time 0)). There was no significant difference in the percentage of *Cox-2* mRNA levels remaining between *AhR^DBD/DBD^* and *AhR^DBD/B6^* fibroblasts. Results are expressed as mean ± SEM of 2–6 independent experiments. (D) *AhR^DBD/DBD^* and *AhR^DBD/B6^* lung fibroblasts were exposed to 1% CSE for 3 hours together with CH-223191 and ActD (1 µg/ml) for the indicated time-points. There was no significant decrease in the percentage of *Cox-2* mRNA remaining when AhR activity is inhibited with CH-223191. Results are expressed as mean ± SEM of normalized *Cox-2* levels and represent results from 3 independent experiments.

After demonstrating that ActD, at the concentrations used in this study (1 µg/ml), prevents IL-1β-induced *Cox-2* mRNA expression (Figure 5A), we performed ActD-chase experiments and quantified the decay in *Cox-2 *mRNA by qRT-PCR analysis. There was a significant decline in *Cox-2* mRNA levels only when *AhR^+/+^* lung fibroblasts were exposed to ActD for 1 or 3 hours (Figure 5B, *black squares*). In contrast, levels of *Cox-2* remained constant and were unaltered in *AhR^−/−^* lung fibroblasts exposed to ActD (Figure 5B, *open diamonds*), suggesting that the AhR destabilizes *Cox-2* mRNA expression. We also utilized *AhR^DBD/DBD^* fibroblasts to evaluate *Cox-2* mRNA stability. There was a rapid and significant decline in steady-state *Cox-2* mRNA levels following ActD exposure for 1, 3 or 6 hours in AhR^DBD/B6^ cells, which express a functional AhR and are thus used as a control to compare with *AhR^DBD/DBD^* cells (Figure 5C, *closed squares*). A parallel and significant decline in *Cox-2 *mRNA was also observed in cells derived from *AhR^DBD/DBD^* mice (Figure 5C, *open diamonds*). There was no significant difference in the percentage of remaining *Cox-2* mRNA between *AhR^DBD/DBD^* and control lung fibroblasts. Inhibition of AhR activity with CH-223191 prevented the significant decline in *Cox-2* mRNA in both control and *AhR^DBD/DBD^* fibroblasts (Figure 5D), supporting that AhR activity is necessary for destabilization of the *Cox-2* transcript. Collectively, these data demonstrate that *Cox-2* mRNA stability is governed by a mechanism independent of the DNA-binding abilities of the AhR.

### The AhR Controls the Nuclear-cytoplasmic Shuttling of HuR in Response to CSE

**Figure 6:**
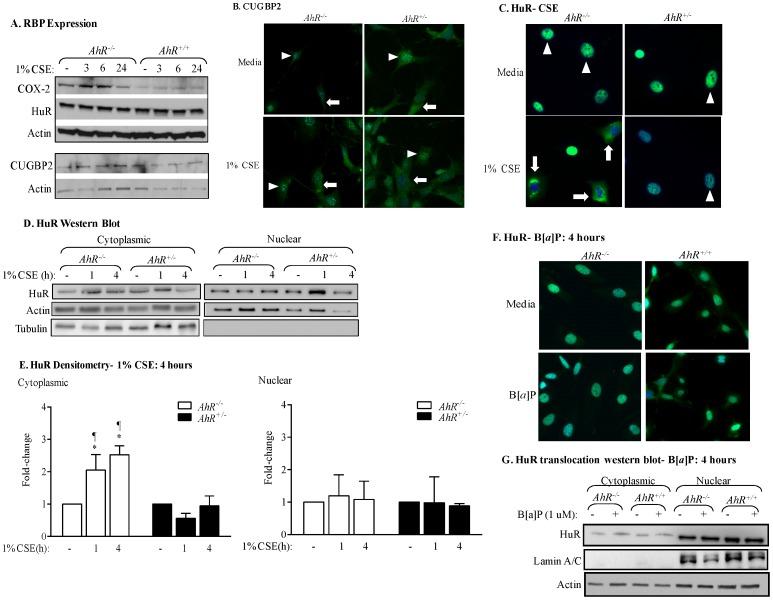
AhR retains HuR in the nucleus in response to CSE but does not contribute to HuR expression. (A) HuR and CUGPB2 are constitutively expressed and are unaffected by AhR expression or CSE exposure. Note that there was an increase in Cox-2 protein in CSE-exposed *AhR^−/−^* fibroblasts but not *AhR^+/+^* cells. (B) *AhR^−/−^* and *AhR^+/−^* lung fibroblasts were exposed to 1% CSE for 4 hours and IF performed for CUGBP2. Nuclei are visualized by Hoechst (blue) and the merged images are shown. CUGBP2 was localized predominantly in the nucleus in *AhR^−/−^* and *AhR^+/−^* fibroblasts (*arrowheads*), although cytoplasmic expression was detectable (*arrows*). Cytoplasmic CUGBP2 increased in both *AhR^−/−^* and *AhR^+/−^* fibroblasts exposed to 1% CSE (*arrows*). (C) In cells treated with media, HuR is predominantly localized in the nucleus both in *AhR^−/−^* and *AhR^+/−^* fibroblasts (*arrowheads*). CSE exposure (1%) for 4 hours in absence of AhR expression (*AhR^−/−^*) induces HuR shuttling from the nucleus to the cytoplasm (*arrows*). When *AhR^+/−^* fibroblasts are challenged with 1% CSE, HuR remains in the nucleus. Results are representative of three independent experiments. (D) There was an increase in cytoplasmic HuR only in the *AhR^−/−^* cells beginning at one hour of exposure and continuing through 4 hours. The purity of the extraction was determined by Tubulin, which was not detectable in the nuclear fraction. Representative western blot is shown. (E) Densitometric analysis of cytoplasmic and nuclear extracts following exposure to CSE: there was a significant increase in cytoplasmic HuR in response to CSE in only *AhR^−/−^* cells (2.5±0.3; *p<0.05 compared to media only; ^¶^p<0.05 compared to respective *AhR^+/−^* fibroblasts exposed to CSE at the indicated time-point). Results are expressed as mean ± SEM of three independent experiments. (F) Classic AhR ligands do not cause cellular HuR redistribution in mouse lung fibroblasts. HuR remained within the nucleus upon exposure to B[*a*]P. Images are representative of two independent experiments. Magnification = 40×. (G) There was no increase in cytoplasmic HuR in *AhR^+/+^* or *AhR^−/−^* cells exposed to B[*a*]P for 4 hours. Representative western blot is shown.

The instability of *Cox-2* mRNA is due to the presence of an ARE in the 3′-untranslated region which facilitates recruitment of RNA-binding proteins [40], including HuR and CUGBP2 [20], [41]. As there is no information on whether RNA-binding protein expression is altered by cigarette smoke exposure or the AhR, we first evaluated the expression of HuR and CUGBP2 by western blot analysis. In both *AhR^−/−^* and *AhR^+/+^* lung fibroblasts, there was similar expression of HuR and CUGBP2 (Figure 6A). There was also no detectible difference in the expression of these RNA-binding proteins upon exposure to 1% CSE in *AhR^−/−^* and *AhR^+/+^* lung cells. Note that there is an increase in COX-2 protein levels only in *AhR^−/−^* cells exposed to CSE (Figure 6A; compared with Figure 1D and [4]).

CUGBP2 and HuR are localized mainly in the nucleus, shuttling to the cytoplasm when appropriately activated [20]. The cytoplasmic localization of HuR and CUGBP2 correlate with their ability to stabilize target mRNAs, including *Cox-2* [21], [22], [23]. CUGBP2 was predominantely nuclear and translocated to the cytoplasm upon exposure to 1% CSE, with little detectible difference between *AhR^−/−^* and *AhR^+/−^* cells (Figure 6B). HuR was also almost entirely restricted to the nucleus (Figure 6C- *arrowheads*). In *AhR^+/−^* cells exposed to 1% CSE, there was no change in the subcellular distribution of HuR (Figure 6C- *arrowheads*- and 6D). In contrast, there was a significant translocation of HuR to the cytoplasm in *AhR^−/−^* cells in response to 1% CSE for either 1 or 4 hours (Figure 6C- *arrows* and 6D and 6E). The relative level of nuclear HuR was not significantly altered by CSE exposure (Figure 6E). AhR activation by the classic ligand B[*a*]P did not alter HuR localization (Figure 6F and 6G).

**Figure 7:**
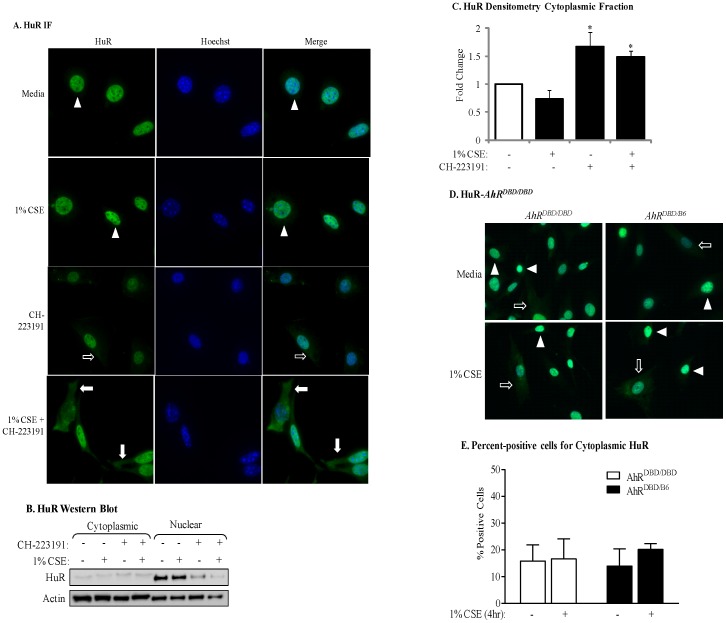
Inhibition of AhR activity in mouse lung fibroblasts by CH-223191 promotes cytoplasmic shuttling of HuR in response to 1% CSE. (A) *AhR^+/−^* mouse lung fibroblasts were pretreated with CH-223191 for one hour prior to being incubated with 1% CSE for an additional 4 hours and IF performed for HuR. HuR localization was restricted to the nucleus in media- and CSE exposed *AhR^+/−^* mouse lung fibroblasts (*arrowheads*). Cells that were treated with CH-223191 exhibited a slight increase in cytoplasmic expression of HuR (*open arrows*) while the majority of HuR remained within the nuclear compartment. When *AhR^+/−^* lung fibroblasts were pretreated with CH-223191, followed by incubation with 1% CSE, there was a pronounced increase in cytoplasmic HuR (*closed arrows*). Magnification = 40×; representative images are shown. (B) Western blot analysis of cytoplasmic and nuclear extracts was performed as described above following exposure of *AhR^+/+^* lung fibroblasts to CSE with our without the AhR antagonist CH-223191 for 4 hours. There was an increase in cytoplasmic HuR in response to CH-223191 as well as CSE plus CH-223191. Representative western blot is shown. (C) There was a significant increase in the level of cytoplasmic HuR in response to CH-223191 (1.67±0.26) as well as 1% CSE plus CH-223191 (1.49±0.1). *p<0.05; results are expressed as mean ± SEM of 2 independent experiments. (D) HuR localization in response to CSE in *AhR^DBD/DBD^* fibroblasts. *AhR^DBD/DBD^* or control fibroblasts were exposed to 1% CSE for 4 hours and HuR localization assessed by IF as described above. HuR remained localized to the nucleus in both *AhR^DBD/DBD^* and *AhR^DBDB6^* cells exposed to CSE. Magnification = 40×. (E) Percent-positive cells: There was no significant difference in the percentage of cells positive for cytoplasmic HuR between *AhR^DBD/DBD^* (16.6±7.5) and *AhR^DBD/B6^* (20±2) cells after exposure to 1% CSE for 4 hours. The number of positive cells was also not different between media or exposure to CSE. Results are expressed as the mean ± SEM.

To then evaluate if AhR activity is necessary to retain HuR in the nucleus, we evaluated HuR localization in AhR-expressing cells which had been pretreated with CH-223191 and then treated with CSE for 4 hours. Exposure of AhR-expressing lung fibroblasts to 1% CSE failed to promote cytoplasmic redistribution of HuR, and HuR remained predominantly nuclear (Figure 7A- *arrowheads*). Inhibition of the AhR by CH-223191 alone resulted in a small but significant increase in cytoplasmic HuR (Figure 7A- *arrows*; Figure 7B and 7C). However, exposure of lung fibroblasts to CH-223191 in conjunction with CSE caused nuclear-cytoplasmic shuttling of HuR (Figure 7A- *arrows*; Figure 7B). This increase in cytoplasmic HuR localization was significant compared to CSE alone (Figure 7C). In both *AhR^DBD/DBD^* and *AhR^DBD/B6^* cells exposed only to media, HuR was mostly nuclear (Figure 7D, *arrowheads*). In response to 1% CSE, HuR remained predominantly nuclear (Figure 7D, *arrowheads*) although there was detectable cytoplasmic HuR that was similar in intensity between *AhR^DBD/DBD^* and *AhR^DBD/B6^* cells, (Figure 7D, *arrows*). The percentage of cells positive for cytoplasmic HuR was not different between *AhR^DBD/DBD^* and *AhR^DBD/B6^* cells (Figure 7E). Collectively, these data show that the AhR controls the nuclear-cytoplasmic shuttling of HuR in response to cigarette smoke by a mechanism that is independent of DNA binding.

**Figure 8:**
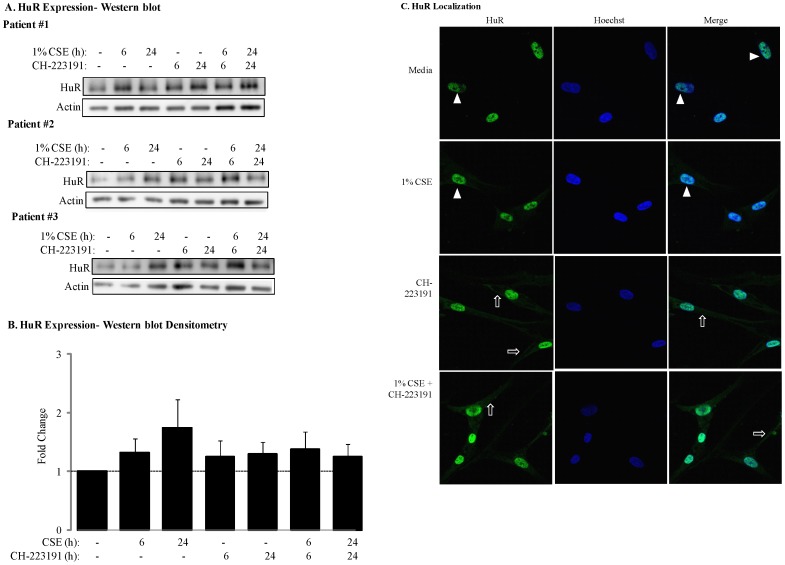
HuR expression and cellular localization in primary human lung fibroblasts after AhR inhibition by CH-223191. (A) Primary human lung fibroblasts from three non-smoking individuals express HuR and the relative expression level was not altered by exposure to CSE. Inhibition of AhR activity with CH-223191 also had no affect on HuR protein levels. (B) Densitometric analysis indicated that there was a slight but not statistically significant change in HuR protein expression in response to CSE for 24 hours. Exposure to the AhR antagonist CH-223191, with or without CSE, also did not significantly alter HuR protein expression. Results are expressed as the mean ± SEM, n = 3 (western blots above). (C). HuR localization was restricted to the nucleus in human lung fibroblasts exposed to 1% CSE (*arrowheads*). Inhibition of AhR activity with CH-223191 slightly increased the amount of HuR in the cytoplasm (*open arrows*). The cytoplasmic redistribution of HuR was further augmented when cells were pretreated with CH-223191 and 1% CSE (*open arrows*). Images are representative of results obtained with lung fibroblasts derived from three different individuals.

HuR expression has been shown in human fetal lung fibroblasts [42] but the expression of HuR in adult lung fibroblasts is not known. Our results now show that HuR is expressed in adult human lung fibroblasts (Figure 8A). Densitometric analysis revealed that there was a slight, but not statistically-significant increase in the expression of HuR upon exposure of human lung fibroblasts to CSE (Figure 8B). Exposure to the AhR antagonist CH-223191, with or without CSE, had no significant effect on HuR expression (Figure 8A and 8B). However, when human lung fibroblasts were exposed to a combination of CSE and CH-223191, there was an increase in cytoplasmic HuR (Figure 8C). Collectively these data strongly support the notion that the AhR is a critical regulator of HuR translocation in pulmonary cells, and whose activity is important in retaining HuR in nucleus.

### siRNA Knockdown of HuR in AhR-deficient Primary Lung Fibroblasts Destabilizes Cox-2 mRNA

**Figure 9:**
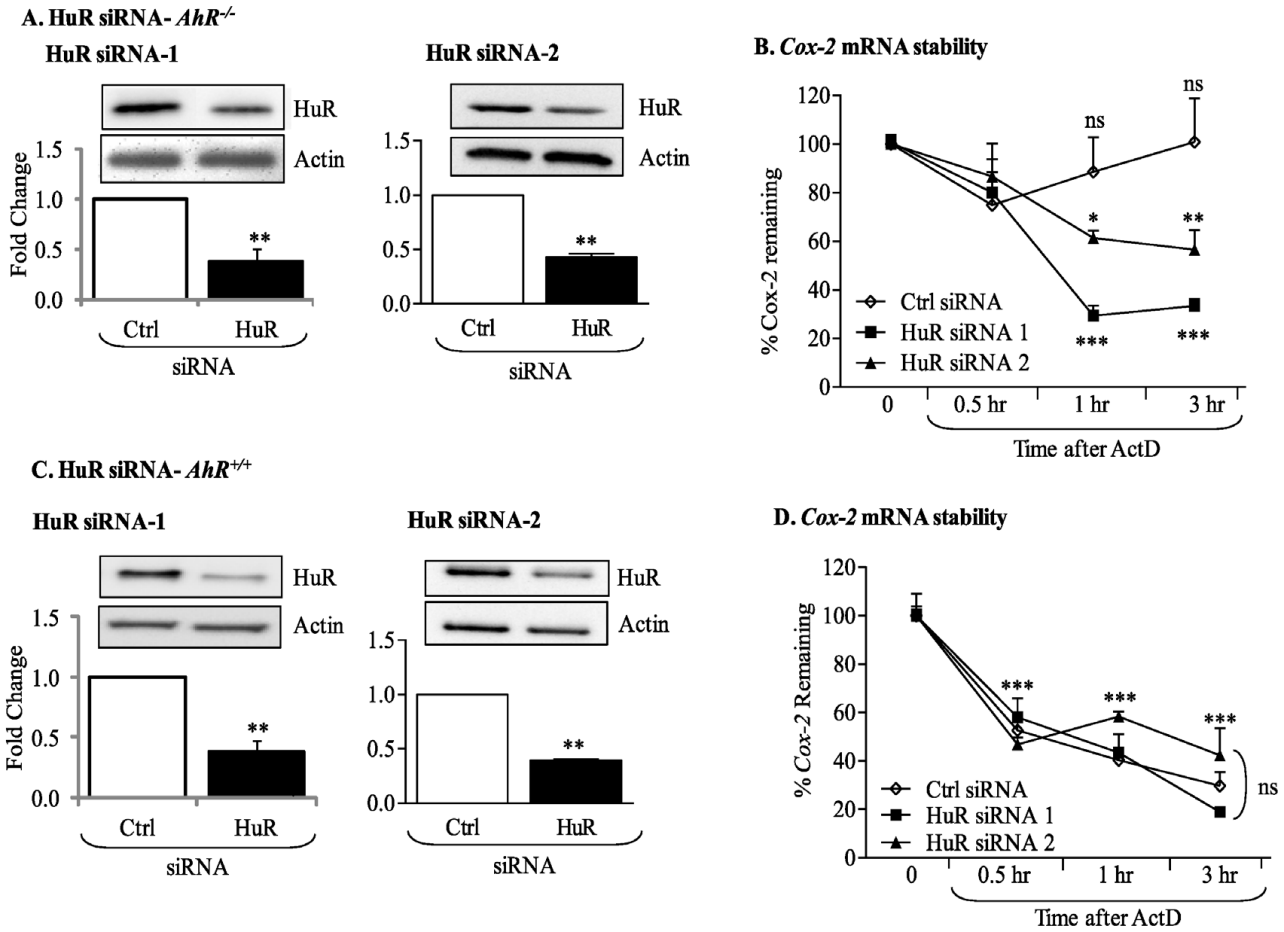
HuR silencing destabilizes *Cox-2* mRNA in CSE-exposed *AhR^−/−^* lung fibroblasts. Fibroblasts were transiently transfected with two siRNA against HuR (siRNA-1 and siRNA-2) or control (Ctrl) siRNA, exposed to CSE and *Cox-2* mRNA stability evaluated by ActD chase experiments. (A) Transfection of *AhR^−/−^* fibroblasts with HuR siRNA significantly reduced HuR protein levels between 50–70%. Results are expressed as the mean ± SEM, n = 3 independent experiments per siRNA construct. (B) *Cox-2* mRNA levels in *AhR^−/−^* fibroblasts transfected with Ctrl siRNA remained stable and did not significantly decline after exposure to ActD (ns = not significant compared to time 0). There was a significant decline in *Cox-2* mRNA when HuR was knocked-down in *AhR^−/−^* cells (**p<0.01 compared to Time 0 of HuR siRNA). This decrease in *Cox-2* mRNA following HuR siRNA-1 and siRNA-2 was significantly lower than the percentage of *Cox-2* remaining in the Time 0 siRNA *AhR^−/−^* fibroblasts (*p<0.05; **p<0.01; ***p<0.001). Results are expressed as the mean ± SEM, n = 2–5 independent experiments. (C) *AhR^+/+^* cells were transfected with two siRNA against HuR (siRNA-1 and siRNA-2); there was a significant reduction in HuR protein levels following knockdown (0.38±0.09- siRNA-1; 0.39±0.012- siRNA-2). Results are expressed as mean ± SEM of three independent experiments. (D) There was a significant decline in *Cox-2* mRNA after exposure to ActD. Knock-down of HuR did not significantly affect the decay of *Cox-2* mRNA levels (***p<0.001 compared to respective Time 0). There was no significant difference in the percentage of *Cox-2 *mRNA remaining between Ctrl, siRNA-1 or siRNA-2 (ns). Results are expressed as mean ± SEM of 2–4 independent experiments.

To confirm the implication of HuR in regulating *Cox-2* stability in *AhR^−/−^* lung fibroblasts, RNA interference was used to decrease HuR protein expression by at least 60% utilizing two different siRNA targeting HuR (Figure 9A and 9C). In *AhR^−/−^* cells, there was no significant change in the steady-state levels of *Cox-2 *mRNA following exposure to ActD for as long as 3 hours (Figure 9B). However, in *AhR^−/−^* lung fibroblasts in which HuR expression was reduced, there was a significant decline in *Cox-2* mRNA expression (siRNA-1 and siRNA-2; Figure 9B). In *AhR^+/+^* cells, HuR knock-down did not affect the decline in Cox-2 mRNA (Figure 9D), demonstrating that HuR is the principle factor that stabilizes *Cox-2 *mRNA in AhR-deficient pulmonary cells.

### Cigarette Smoke Exposure Increases Pulmonary COX-2 Protein Expression and Nuclear-Cytoplasmic Shuttling of HuR in AhR^−/−^ Mice

**Figure 10:**
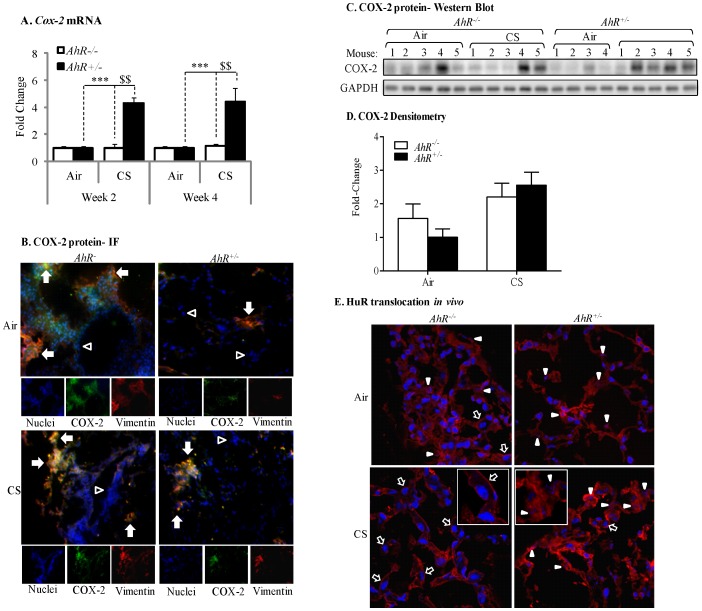
Cigarette smoke induction of pulmonary COX-2 protein expression in *AhR*-deficient mice is associated with increased cytoplasmic HuR. (A) There was a significant induction of *Cox-2* mRNA in the lungs of *AhR^+/−^* mice exposed to cigarette smoke for 2 (4.3±0.4) or 4 (4.4±1.0) weeks compared to air-exposed mice (***p<0.001). This induction in *Cox-2* mRNA was significantly greater than CS-exposed *AhR^−/−^* mice (^$$^p<0.01). There was no induction of *Cox-2 *mRNA in the lungs of *AhR^−/−^* mice exposed to CS. Results are expressed as the mean ± SEM, n = 4–5 mice per group. (B) There is an increase in pulmonary COX-2 levels in response to CS in lung fibroblasts (orange/yellow color- *arrows*). Note that vimentin-negative cells (Hoechst- *blue color*- only) do not increase COX-2 in response to CS and likely reflect epithelial cells. Magnification = 40× and images are representative of COX-2 protein expression in the lungs of three different mice of each genotype. (C) COX-2 induction occurred in CS-exposed *AhR^−/−^* and *AhR^+/−^* mice. (D) Densitometric analysis revealed that CS elevates COX-2 in *AhR^−/−^* (2.2±0.4) and *AhR^+/−^* (2.5±0.39) mice. Results are expressed as the mean ± SEM, n = 7–8 mice per group. Representative western blot of 4–5 individual mice is shown. (E) The lungs of *AhR^+/−^* mice exposed to CS for 2 weeks exhibited some cytoplasmic translocation (*open arrow*) although HuR remained predominantly nuclear (pink color-*arrowheads*). There was a pronounced redistribution of HuR to the cytoplasm in the lungs of *AhR^−/−^* mice exposed to CS (*open arrows*). Inset shows enlarged region depicting difference in HuR localization; note the clearly visible nuclei (blue) in the AhR^−/−^ lung. Images are representative of HuR in the lungs of three different mice of each genotype.

To confirm our *in vitro* findings on the AhR regulation of COX-2 protein levels via nuclear retention of HuR, we utilized our preclinical murine model and exposed *AhR^−/−^* and *AhR^+/−^* mice to cigarette smoke. There was a significant induction in *Cox-2 *mRNA only in the lungs of *AhR^+/−^* mice following cigarette smoke exposure for 2 or 4 weeks compared to animals exposed only to room air (Figure 10A). There was no increase in *Cox-2* mRNA in the lungs of *AhR^−/−^* mice (Figure 10A) at either time-point. Because there was no difference in the induction of *Cox-2 *mRNA between 2- and 4-week exposures, only the 2-week exposure regime was analyzed for COX-2 protein expression and HuR localization.

Despite the lack of *Cox-2* mRNA induction in *AhR^−/−^* mice exposed to cigarette smoke, there was an increase in pulmonary COX-2 protein expression (Figure 10B) and a trend towards higher basal COX-2 protein in the lungs of *AhR^−/−^* mice (Figure 10C and 10D). There is also COX-2 protein expression in the lungs of *AhR^+/−^* mice exposed to cigarette smoke (Figure 10C and 10D). Double-immunofluorescence utilizing antibodies for both COX-2 and vimentin (a fibroblast marker) indicated that COX-2 protein was readily evident in lung fibroblasts (Figure 10B, *arrows*- orange/yellow color), with stronger fluorescence occurring in the lungs of AhR^−/−^ mice. There were also abundant vimentin-negative cells without COX-2 expression (blue color-nuclei only) and likely represent epithelial cells (Figure 10B, *open arrowheads*). In the lungs of air-exposed mice, HuR was abundantly expressed and was localized predominantly in the nucleus (Figure 10E, *arrowheads*) although cytoplasmic localization was evident. In the lungs of *AhR^+/−^* mice exposed to cigarette smoke for 2 weeks, there was some translocation of HuR to the cytoplasm (Figure 10E, *open arrow*). There was also considerable nuclear HuR remaining (*arrowheads*). In stark contrast, redistribution of HuR to the cytoplasm was unmistakable in the lungs of *AhR^−/−^* mice exposed to cigarette smoke (Figure 10E, *open arrows; inset*), indicating *in vivo* that the AhR controls the cellular localization of HuR in response to respiratory toxicants. When considered together, our *in vivo* and *in vitro* data show for the first time that the AhR is a potent suppressor of cigarette smoke-induced pulmonary COX-2 protein due to post-transcriptional regulatory mechanisms that prevent the cytoplasmic translocation of the RNA-binding protein HuR.

## Discussion

The AhR was discovered nearly four decades ago as the receptor responsible for the induction of aryl hydrocarbon hydroxylase (CYP1A1) activity in response to the potent anthropogenic ligand dioxin [43]. Although it is generally accepted that the majority of deleterious effects of dioxin arise from dioxin binding to the AhR and subsequent alterations in gene expression patterns [44], [45], one of the eminent unresolved questions is why organisms would possess a receptor for dioxin at all [46]. The fact though that the AhR is ubiquitously expressed in mammals, being present in all major cell types in humans [47], and is highly-conserved throughout evolution suggests a prominent role for this receptor in mammalian physiology [48]. Early pioneering studies using *AhR*-null mice not only revealed that the AhR is responsible for dioxin toxicity, but have also implicated the AhR in cell proliferation, differentiation, migration, development, tissue homeostasis and vasculogenesis [48], [49]. We have published that low/absent AhR levels increase inflammation and structural cell apoptosis [4], [24], findings which argue for a prominent role of the AhR in normal physiology. In our current study, we sought to identify the mechanism by which the AhR prevents inflammatory protein expression and report that AhR-dependent retention of nuclear HuR suppresses COX-2 expression by a post-transcriptional mechanism.

One of the most significant findings from this study is that the AhR suppresses COX-2 protein expression in the absence of a functional DNA-binding domain. This suggests that the AhR suppresses inflammation by a mechanism that is independent of its transcriptional abilities. In its paradigm as ligand-activated transcription factor, the AhR utilizes a classic mechanism of action involving nuclear translocation and binding to specific DNA recognition sequences to activate genes associated with toxicological outcomes. This canonical AhR pathway is believed to mediate the toxicity of dioxin and similar compounds due to DRE-mediated upregulation of phase I and II drug-metabolizing enzymes (*e.g*. CYP1A1 and CYP1B1). However, recent evidence indicates that the AhR has a separate mode of action beyond direct transcriptional regulation [50], thereby representing an AhR pathway that is distinct from the one associated with dioxin-induced toxicity. Others have also shown that this non-canonical anti-inflammatory pathway involves AhR nuclear translocation but not DNA binding [51], [52], suggesting that some AhR activity may be required to effectively prevent inflammation. When we used CH-223191, an AhR antagonist that blocks ligand binding and subsequent translocation to the nucleus [37], we observed a potentiation of cigarette smoke-induction of COX-2 protein expression (Figure 3), signifying that some AhR activity is necessary to keep inflammatory protein levels under control. It would not be unreasonable to assume that endogenous AhR ligands present in organs such as the lung [53] maintain constitutive AhR activity at levels that do not cause alterations in gene expression but are sufficient to prevent an exaggerated inflammatory response.

Such selective modulation of AhR activity could be why activation of the AhR by CSE repressed COX-2 expression, whereas classic AhR ligands such as TCDD increase COX-2 protein levels [11], [12]. Both CSE and B[*a*]P increased AhR activation in lung fibroblasts, as evaluated by *Cyp1a1* mRNA induction (Figure 1), suggesting that the incongruity of results between CSE and B[*a*]P is not due to inability of CSE to activate the AhR. Discrepancy in physiological responses to AhR ligands have been observed elsewhere, including murine models of multiple sclerosis. Here, activation of the AhR by either TCDD or ITE suppresses experimental autoimmune encephalomyelitis (EAE) [54], [55] yet is enhanced by FICZ [56]. Moreover, both ITE and TCDD elicit the same pattern of AhR-dependent gene expression [57], yet ITE does not cause toxicological outcomes associated with dioxin exposure [58], suggesting that their divergent mechanisms of action may be independent of classic AhR activation. The AhR binds to a structurally-diverse array of ligands, and it has been postulated that differential binding to the AhR may contribute to divergence in overall functionality [59]. The high plasticity of ligand effects on signaling pathways, including ligand-dependent differences in co-factor recruitment to target genes [60], may account for some of this variance [61]. Our results show that suppression of COX-2 protein in response to CSE is due to the AhR, concurrent with HuR nuclear retention (Figure 6), both of which did not occur with classic AhR ligands, suggests divergent mechanisms of COX-2 regulation by the AhR.

Despite our results showing lack of *Cox-2* mRNA induction in absence of AhR expression, there is a profound increase in COX-2 protein (Figure 1) [4], implicating post-transcriptional mechanisms as the way in which the AhR prevents COX-2 expression. Mammalian cells have evolved post-transcriptional mechanisms that further control inflammatory protein levels [40]. Post-transcriptional control is accomplished by regulating nuclear export, cytoplasmic localization, translation initiation and mRNA decay [62], the latter being determined by the presence of the ARE in the 3′-UTR of mature mRNA [63]. Many transiently-expresses cytokines, growth factors and other mediators, including *Cox-2*, contain AREs and whose mRNA is rapidly destabilized [17], [64], [65]. Our results demonstrate that CSE-induction of *Cox-2* mRNA in AhR-expressing cells was transient, and returned to baseline by 6 hours, indicative of rapid mRNA decay [4]. Using ActD, an inhibitor of RNA synthesis [33], [66], we show that when the AhR is expressed, *Cox-2* mRNA is rapidly degraded (Figure 5). Thus, post-transcriptional regulation of protein expression by the AhR may be an important adaptive mechanism to control cellular perturbations caused by environmental stress.

Stress responses can profoundly affect mRNA stability via the concerted efforts of numerous RNA-binding proteins including CUGBP2, TTP and HuR [67], all of which can play a role in regulating *Cox-2* expression [19], [68], [69], [70]. Both CUGBP2 and HuR are nuclear proteins, undergoing translocation to the cytoplasm in response to a variety of stress conditions, including γ-irradiation (IR) [19], reactive oxygen species [71] and ATP depletion [72]. This nuclear-cytoplasmic shuttling is believed to provide protection against *Cox-2* mRNA degradation [19], [21]. The transcriptional regulation of HuR is virtually unknown [73] and there is no information on whether cigarette smoke alters the expression or localization of RNA-binding proteins, including CUGBP2 and HuR. Therefore, our data are the first to show that neither cigarette smoke nor AhR expression alters the expression levels of CUGBP2 or HuR (Figures 6 and 8). However, the AhR profoundly controls the nuclear levels of HuR in response to CSE. HuR translocation from the nucleus to the cytoplasm is critical to its ability to stabilize target mRNA [21], [74], [75]. This may be why in *AhR^+/+^* cells, where HuR remains within the nucleus, HuR knock-down had no effect on *Cox-2* mRNA stability (Figure 9). Results in C5N cells, a mouse keratinocyte cell line with exclusive nuclear HuR [76] support this notion, as reduction in HuR expression had no effect on ornithine decarboxylase (ODC) mRNA stability [76]. However in *AhR^−/−^* cells whereby HuR translocates to the cytoplasm, HuR was a key factor involved in *Cox-2* mRNA stability, as siRNA-knockdown resulted in enhanced *Cox-2* mRNA degradation. Our results support that retention of nuclear HuR is an important feature in the destabilization of *Cox-2* mRNA by the AhR. In addition to *Cox-2*, HuR has thousands of target genes [77] and stabilizes mRNAs that encode proteins associated with a variety of cellular functions including cell cycle (cyclin D1), proliferation (c-Fos), apoptosis (Bcl-2, cytochrome C) and inflammation (TLR4, IL-6, IL-8) [78]. The AhR regulation of these functions is established [4], [5], [24], [79], [80] opening the possibility that AhR retention of nuclear HuR may have important implications for the regulation of genes beyond the control of *Cox-2*.

Our results are also the first to show *in vivo* evidence of pulmonary HuR translocation in response to cigarette smoke (Figure 10). In the lungs of *AhR^−/−^* mice, there was no *Cox-2* mRNA induction despite concordant COX-2 protein and profound cytoplasmic HuR. It was surprising to note considerable levels of HuR in the cytoplasm of pulmonary cells without smoke exposure. Cytoplasmic HuR has been reported in the lungs of adult A/J mice [81], consistent with our data, and HuR expression is required for proper lung development [82]. It may be that in the lung, an organ continuously exposed to the environment and one that is highly susceptible to oxidative damage, a constitutive level of cytoplasmic HuR is required to ensure optimum immunological responsiveness.

Although our results reveal a novel pathway in which the AhR regulates COX-2 protein expression by controlling the cellular localization of HuR, it remains to be established precisely how the AhR retains HuR in the nucleus. Our finding that the AhR regulates HuR localization in response to CSE, but not B[*a*]P (Figure 6) indicates divergent mechanism of AhR activation in maintaining HuR localization despite the ability of both to cause *Cyp1a1* mRNA induction (Figure 1). It also indicates that B[*a*]P, which is present in cigarette smoke [83], [84], is not the component(s) causing HuR translocation to the cytoplasm in the absence of AhR expression. Cigarette smoke is a complex mixture, containing more than 4800 compounds including metals, oxidants and free radicals [85], the latter of which are a potent source of oxidative stress. Given that the AhR protects against oxidative damage due to smoke exposure [24], [86], it reasonable to speculate that the high oxidant conditions exerted by cigarette smoke (an estimated 10^17^ oxidant molecules per puff) [85] contributes to HuR translocation in the absence of AhR expression. AhR activity was required to retain HuR within the nucleus, but did not require DNA-binding (Figure 7). It has been speculated that the DRE-independent anti-inflammatory abilities of the AhR may involve multiple protein-protein interactions [51]. The AhR localizes to the nucleus in the absence of exogenous ligand, a cellular phenomenon that depends on cell-cell contact [87], [88]. Adherent cells grown to sub-confluence, methodologically similar to the experiments conducted here, exhibit both cytoplasmic and nuclear AhR [87], making interaction with AhR and HuR within the nucleus a plausible assumption. Thus, while there is no known physical association between AhR and HuR, it is interesting to speculate that the AhR may interact with HuR to prevent its nuclear export, a notion we are actively pursuing.

**Figure 11:**
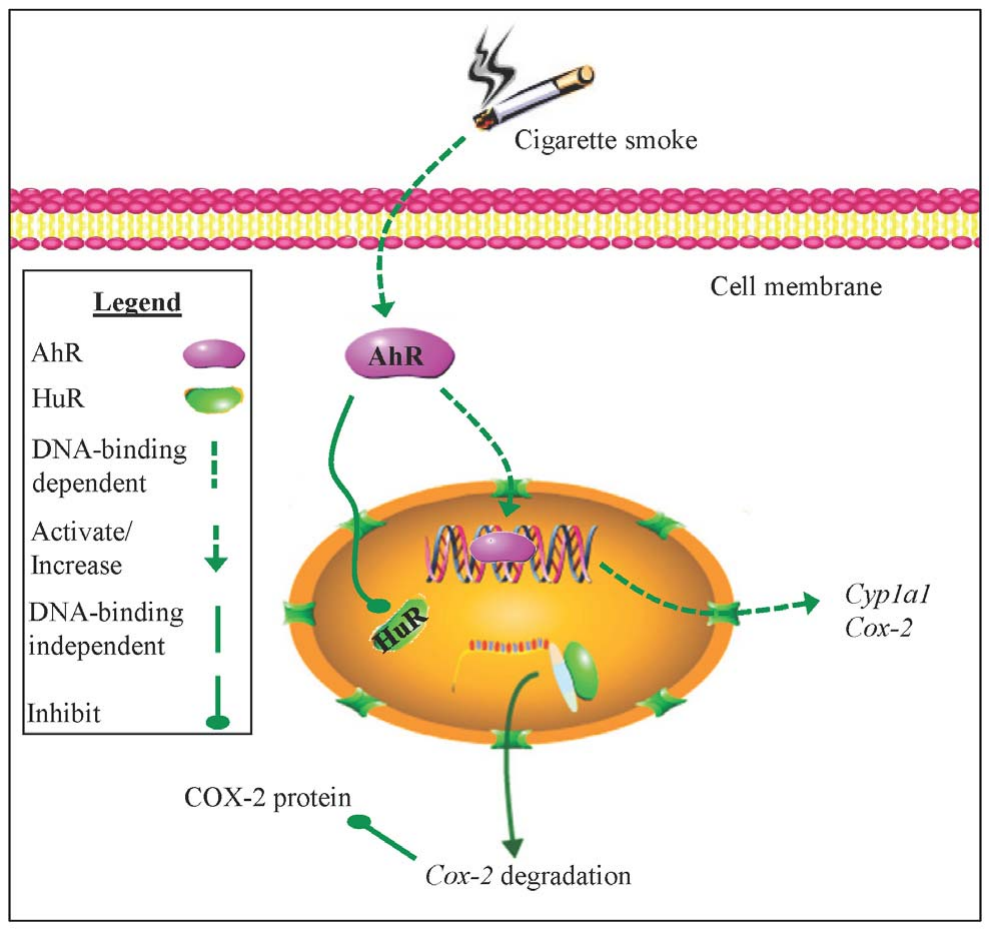
Schematic depiction of AhR-dependent attenuation of COX-2 protein by nuclear retention of HuR. Cigarette smoke activates the AhR, which translocates to the nucleus and binds DNA, resulting in an increase in AhR-dependent gene transcription (e.g. *Cyp1A1* and *Cox-2* mRNA). The AhR also rapidly destabilizes *Cox-2* mRNA by retaining HuR within the nucleus, suppressing an exaggerated increase in COX-2 protein expression. The AhR retention of nuclear HuR and subsequent suppression of COX-2 protein does not involve classic AhR:DNA binding but the mechanism by which AhR retains HuR within the nucleus is not known.

It is believed that the AhR plays an important role in physiology independent of its ability to respond to dioxin. Our previous work highlights the AhR as a key anti-inflammatory protein by an unknown mechanism [4], [5]. Herein, we report that the AhR suppresses COX-2 protein expression in response to cigarette smoke by enhancing *Cox-2* mRNA decay, a fundamental process that does not involve classic DRE-mediated transcription (Figure 11). We show for the first time that the AhR controls HuR localization, an RNA-binding protein critical in stabilizing *Cox-2 *mRNA expression levels. A DRE-independent AhR pathway has the potential to be exploited as an anti-inflammatory target, a notion made increasingly feasible with the characterization of selective AhR modulators, a class of AhR ligands without dioxin-associated toxicity [89]. Collectively, these results establish that the function of AhR extends beyond its ability to respond to man-made toxicants and solidifies the AhR as part of a regulatory pathway that suppresses inflammatory protein expression.
